# Supplementary material for: Kidney function on admission predicts in-hospital mortality in COVID-19
Source: PLoS One. 2020 Sep 3;15(9):e0238680. doi: 10.1371/journal.pone.0238680 (PMC7470363; doi:10.1371/journal.pone.0238680)
Supplement: Attachment — Submitted filename: Response to Reviews.docx [file pone.0238680.s002.docx]

Section Editor

August 13, 2020

Chiara Lazzeri

Academic Editor

PLOS ONE

Dear Editor,

First of all, we would like to thank you very much for your valuable review, which gave us the opportunity to improve our manuscript (PONE-D-20-18434).

We carefully read all suggestions made by the Editorial Board and the reviewers. Below, you can find our responses to these comments. We made the necessary changes suggested in these comments.

We sincerely appreciate the useful and expert suggestions that helped us to prepare the revised manuscript. We thank you for your reconsideration.

Yours truly,

Nurhan Seyahi, MD

**Editorial Review Board Comments:**

1- Thank you for including your ethics statement: 'The study protocol was approved by the local medical ethical committee (approval no: 2020-56318) and the Scientific Committee of the Ministry of Health (approval no: 2020-05-07T13_09_11).'
(a) Please amend your current ethics statement to include the full name of the ethics committee that approved your specific study. 
(b) Once you have amended this/these statement(s) in the Methods section of the manuscript, please add the same text to the “Ethics Statement” field of the submission form (via “Edit Submission”).
For additional information about PLOS ONE ethical requirements for human subjects research, please refer to <http://journals.plos.org/plosone/s/submission-guidelines#loc-human-subjects-research>.

**Response:** We made the suggested changes.

2. In the ethics statement in the manuscript and in the online submission form, please provide additional information about the patient records used in your retrospective study, including: a) whether all data were fully anonymized before you accessed them; b) the date range (month and year) during which patients' medical records were accessed; and c) the source of the medical records analyzed in this work (e.g. hospital, institution or medical center name). If the ethics committee waived the requirement for informed consent, please include this information.

**Response:** a) We anonymized the database during our analysis. b) We accessed the database between March 15 and May 1, 2020. c) The source of medical records was ISHOP (Istanbul University-Cerrahpasa Hospital Automation Program) electronic database system. d) The ethics committee waived the requirement for informed consent.

3. We suggest you thoroughly copyedit your manuscript for language usage, spelling, and grammar. If you do not know anyone who can help you do this, you may wish to consider employing a professional scientific editing service.  

Whilst you may use any professional scientific editing service of your choice, PLOS has partnered with both American Journal Experts (AJE) and Editage to provide discounted services to PLOS authors. Both organizations have experience helping authors meet PLOS guidelines and can provide language editing, translation, manuscript formatting, and figure formatting to ensure your manuscript meets our submission guidelines. To take advantage of our partnership with AJE, visit the AJE website ([http://learn.aje.com/plos/](about:blank)) for a 15% discount off AJE services. To take advantage of our partnership with Editage, visit the Editage website (www.editage.com) and enter referral code PLOSEDIT for a 15% discount off Editage services.  If the PLOS editorial team finds any language issues in text that either AJE or Editage has edited, the service provider will re-edit the text for free.

Upon resubmission, please provide the following:

- The name of the colleague or the details of the professional service that edited your manuscript
- A copy of your manuscript showing your changes by either highlighting them or using track changes (uploaded as a *supporting information* file)
- A clean copy of the edited manuscript (uploaded as the new *manuscript* file)

**Response:** The manuscript was edited by Edvard Tony Karakas from Daria Consulting, Turkey.

**Response to Reviewer**

Reviewer #1: Although this reviewer warmly welcomes this manuscript, some issues should be addressed:

1. The rationale for the study is unclear as the introduction is a bit confusing, comprising several pieces of apparently unlinked information. A more integrated appraisal of the relevant literature would be appropriate to provide the context for the study.

**Response:** We are sorry for this inconvenience. Apparently, a part of the introduction was deleted inadvertently during the submission. Following your comment, we became aware of this error and corrected the relevant section.

2. Figures were not available.

**Response:** We are sorry for this inconvenience. We believe that the figures were deleted because of a technical issue during the submission. We checked that they are correctly incorporated to the revised manuscript.

3. The following reports should be mentioned:
J Clin Med. 2020 Jun 3;9(6):E1718; doi: 10.3390/jcm9061718; PMID: 32503180.
J Clin Med. 2020;9(5):1529. doi: 10.3390/jcm9051529. PMID: 32438617.

**Response:** Thank you for providing us those important studies. We cited them in the revised manuscript.

4. Values of systolic and diastolic blood pressure should be included in the analysis.

**Response:** We collected the systolic and diastolic blood pressure records from our hospital electronic database and we made the necessary analysis in the revised manuscript.

5. The key role of endothelial dysfunction in the pathophysiology of kidney injury in COVID-19 (J Clin Med. 2020 May 11;9(5):1417; doi: 10.3390/jcm9051417; PMID: 32403217) should be better discussed.

**Response:** Thank you for providing us this important paper. We extended the Discussion section with the help of this paper.

6. English language (syntax, grammar, correct choice of words, correct use of adjectives and adverbs) should be substantially improved throughout the text. Professional assistance should be sought.

**Response:** With the assistance of a professional service, we revised the manuscript regarding language issues.
